# *SIRT1* Allele Frequencies in Depressed Patients of European Descent in Russia

**DOI:** 10.3389/fgene.2018.00686

**Published:** 2019-01-04

**Authors:** Lyubomir I. Aftanas, Maksim S. Anisimenko, Darya A. Berdyugina, Aleksandr Yu. Garanin, Vladimir N. Maximov, Mikhail I. Voevoda, Natalya M. Vyalova, Nikolay A. Bokhan, Svetlana A. Ivanova, Konstantin V. Danilenko, Sergei P. Kovalenko

**Affiliations:** ^1^Institute of Physiology and Basic Medicine, Novosibirsk, Russia; ^2^Institute of Medicine and Psychology, Novosibirsk State University, Novosibirsk, Russia; ^3^Institute of Cytology and Genetics, Novosibirsk, Russia; ^4^Tomsk National Research Medical Center of the Russian Academy of Sciences, Mental Health Research Institute, Tomsk, Russia; ^5^Department of Psychotherapy and Psychological Counseling, National Research Tomsk State University, Tomsk, Russia; ^6^Institute of Non-Destructive Testing, National Research Tomsk Polytechnic University, Tomsk, Russia

**Keywords:** *SIRT1* gene, SNP, depressive disorders, population, European descent

## Abstract

Depressive disorder (DD) is a widespread mental disorder. Although DD is to some extent inherited, the genes contributing to the risk of this disorder and its genetic mechanisms remain poorly understood. A recent large-scale genome-wide association Chinese study revealed a strong association between the *SIRT1* gene variants and DD. The aim of this study was to analyze the occurrence of heterozygote carriers and search for rare SNP variants of the *SIRT1* gene in a cohort of DD patients as compared with a cohort of randomly selected members of the Russian population. The complete coding sequences of the *SIRT1* gene from 1024 DNA samples from the general Russian population and from 244 samples from patients with DD were analyzed using targeted sequencing. Four new genetic variants of the *SIRT1* were discovered. While no significant differences in the allele frequencies were found between the DD patients and the general population, differences between the frequencies of homozygote carriers of specific alleles and occurrences of heterozygous were found to be significant for rs2236318 (*P* < 0.0001), and putatively, rs7896005 (*P* < 0.05), and rs36107781 (*P* < 0.05). The study found for the first time that two new SNPs (i.e., 10:69665829 and 10:69665971) along with recently reported ones (rs773025707 and rs34701705), are putatively associated with DD. The revealed DD-associated *SIRT1* SNPs might confer susceptibility to this disorder in Russian population of European descent.

## Introduction

Depressive disorder (DD or unipolar depression, which includes Major Depressive Disorder [MDD] and dysthymia) is a widespread mental disorder, with heritability as a one of the risk factors. Multiple genes are enganged in development of depression, however, the “genetic architecture” of DD has yet to be elucidated (Peterson et al., 2017).

Recent studies have shown that dysregulation of the function of intracellular proteins has an important role in the pathogenesis of a wide range of mental disorders. The proteins involved in neurobiological processes are believed to be potential targets for pharmacotherapy and/or new markers for diagnostics and the predicted course of affective disorders (Dunman and Violeti, 2013; Losenkov et al., 2014, 2016).

Silent information regulator-1 (*SIRT1*) deacetylase, a protein with NAD(+)**-**dependent deacetylation activity, is a member of the sirtuin protein family. The substrates of *SIRT1* are histones, transcription factors and some other DNA-binding proteins (Hsu et al., 2016). Deacetylation changes the affinity of proteins to DNA, leading to the alteration of the transcription activity of various genes and, consequently, a variety of phenotypic effects.

Silent information regulator-1 has been shown to mediate various cellular processes including inflammation, mitochondrial biogenesis, cell growth, apoptosis, as well as cell senescence and consequent aging (Donmez and Outeiro, 2013; Wang, 2014; Ng et al., 2015; Tang et al., 2018). The levels of *SIRT1* are decreased in both transcriptional and post-transcriptional conditions during aging, accompanied by attenuated mitochondrial biogenesis, an important component of aging-related diseases (Yoshizaki et al., 2010; Rahman and Islam, 2011; Yuan et al., 2016).

Silent information regulator-1 was indexed to be involved into early neuron development, synaptic plasticity, and memory regulation mechanisms (Gao et al., 2010) as well as activate MAO-A in the brain to mediate anxiety and exploratory drive (Libert et al., 2011). It binds to and transcriptionally regulates the gene locus encoding the G protein-coupled receptor GPR50 in the brain, identified as a genetic risk factor for bipolar disorder and DD in women Joerg et al., 2015).

It has been recently evidenced that *SIRT1* participates in regulating inflammatory responses via expression of interleukin-6 (Tang et al., 2017). Given that down-regulation of *SIRT1* promotes the secretion of IL-6 (Volonte et al., 2015), the engagement of *SIRT1* into pathophysiology of DD may be implemented through neuroinflammatory pathways (Ng et al., 2015). Possible roles of mitochondrial dysfunctions and *SIRT1* in DD pathogenesis are also being currently under the scope (Kambe and Miyata, 2017).

Silent information regulator-1 is coded by the *SIRT1* gene. All of the above on *SIRT1* protein as well as recently obtained data on significant down-regulation of the peripheral blood mRNA-based *SIRT1* expression in the patients with DD (Luo and Zhang, 2016) strengthen the suggestion that *SIRT1* may be regarded as a putative risk gene of DD.

As for the genetic facet of the *SIRT1* world, data on genetic variations in *SIRT1* and their associations with DD are scarce and contradictory. An association between rs10997875 in *SIRT1* gene and DD was found in the Japanese population. Thus, the authors did not find any association between *SIRT1* gene and SSRI therapeutic response in DD in the allele/genotype analysis or haplotype analysis (Kishi et al., 2010).

A low-coverage whole-genome analysis of a large cohort of Chinese patients with DD revealed a substantial difference in the frequencies of two SNPs (rs12415800 and rs35936514) between patients with DD and the general population. One of those SNPs (rs12415800) was mapped to the *SIRT1* locus (CONVERGE Consortium, 2015). Data from the study of Finnish population evidenced associations of the *SIRT1* rs3758391 T allele with DD, odds ratio = 1.19 (Kovanen et al., 2015).

A large study based on the DNA from individuals of European descent participating in the consumer genomics company 23andMe’s optional research initiative revealed 15 new loci associated with DD. Meanwhile, the authors were unable to replicate the strong association for SNPs in the *SIRT1* gene found for the Chinese population and only a modest association for rs12415800 was reported (Hyde et al., 2016).

The newest data and meta-analysis on *SIRT1* polymorphism and clinical depression in a Han Chinese population did not find any significant difference in allelic distribution of the rs3758391 between controls and patients with DD (Tang et al., 2018). However, the authors evidenced a wide divergence of the T allele frequency of rs3758391 among different ethnic populations (85% in Han Chinese vs. 27% in Caucasians), suggesting that rs3758391 polymorphism could be ethnicity-associated. Finally, the study showed a significant association of *SIRT1* expression with rs3758391 in the occipital cortex, involved into pathophysiology of DD. Namely, for this brain region, the carriers of the CC genotype manifested significantly lower levels of *SIRT1* expression than those with the TT genotype (Tang et al., 2018).

While there are promising data on *SIRT1* as a novel DD risk gene, association of its allelic variants with DD still remains unclear. Moreover, the majority of publications are focused on the analysis of the most frequent allelic variants, whereas potential associations with rare allelic variants ate still unknown. Moreover, the occurrences of heterozygote/homozygote carriers were not analyzed in details. In view of the above, the objective of the study was to identify rare SNP variants of the *SIRT1* gene and analyze occurrences of heterozygote/homozygote carriers in a cohort of randomly selected members of the Russian population (Novosibirsk city) in comparison with a cohort of DD patients.

## Materials and Methods

### Participants

One thousand and twenty-four samples were chosen (according to the list of random numbers generated by SPSS software) from a large collection of genomic DNA obtained from 45 to 69-year-olds of European descent living in Novosibirsk city in 2003–2005 (HAPIEE epidemiological study^1^). This derived population sample was not analyzed for the presence of psychopathologies.

For the depression sample, DNA was derived from the patients with depression hospitalized in psychiatric units of the Institute of Physiology and Basic Medicine, Novosibirsk and Mental Health Research Institute, Tomsk (a city in close proximity to Novosibirsk) in 2009–2017. The selection criteria included a DSM-IV diagnosis of unipolar depression (MDD or chronic depression) and European ancestry. The diagnoses were assessed by psychiatrists using structured interview – M.I.N.I. (Sheehan et al., 1998; in 71% of cases) or SCID (First et al., 1997; in 29% of cases). All studies were approved by local Ethics Committees, and the signed informed consent included the permission from the patients to perform a genetic analysis of their DNA samples.

The blood was sampled in tubes and frozen at -20°C. Genomic DNA was extracted by the phenol-chloroform method.

### NGS Analysis of *SIRT1* Gene

Nine exons of the *SIRT1* gene were analyzed by targeted sequencing. All exons with their adjacent intron (20–50 bp) were covered by 19 amplicons (225–275 bp). The DNA fragment 5′ adjacent to the *SIRT1* gene containing the SNP named rs12415800 was sequenced as well. Primers were designed using AmpliSeq Designer and Primer 3 software. Twenty selected fragments were amplified in two pools. 20 ng of genomic DNA was used for each reaction containing SuperHotTaq DNA polymerase (Bioron, Germany), 3.0 mM MgCl_2_, 0.2 mM dNTPs, 0.4 μM of each primer. All reactions were performed in 25 μl volume with the following cycling program: 95^o^C for 2 min, then 25 cycles at 95^o^C for 15 s, 60^o^C for 30 s, and 72^o^C for 40 s.

For each DNA sample, 25 μl of the two PCR reactions were combined and purified by Agencourt AMPure XP (Beckman Coulter, Pasadena, CA, United States). The resulting amplicons were ligated with the barcodes and A/P1 adapters. A and P1 adapters were used in the additional PCR round for the enrichment. Qubit dsDNA HS Assay kit was used for resulting fragments concentration measurement on a Qubit 3.0 fluorimeter (Thermosphere, United States). The lengths of the fragments were determined using a High Sensitivity DNA kit (Agilent Technologies, Santa Clara, CA, United States) and the 2100 Bioanalyzer (Agilent Technologies). Normalized libraries were used in emulsion PCR with the Ion PGM Hi-Q OT2 kit and sequenced on Ion Torrent PGM (with Ion PGM HI-Q sequencing kit) according to the manufacturer’s instructions. To reduce the number of samples, two samples were pooled for each barcode.

PRINSEQ (Schmieder and Edwards, 2011) was used for quality control, filtering, and trimming the reads. All reads were mapped to the human genome reference GRCh37/hg19 using BWA-MEM v. 0.7.5 (Li and Durbin, 2009). SAM tools (Li et al., 2009) were used for SNVs detection. Selected variants were manually examined with Integrative Genomic Viewer (Robinson et al., 2011).

All SNP variants were named according to the guidelines of the Human Genome Variation Society, HGVS (Den Dunnen and Antonarakis, 2000). Nucleotide and amino acid numbering were based on GenBank reference sequences Nc_000010.10 and NP_036370.2, respectively. If an SNP was found in the pooled sample, samples from the pool were sequenced individually on an ABI 3130 Genetic Analyzer. The most frequent SNPs were individually analyzed by allele-specific real-time PCR.

Statistical analyses of differences in allele frequencies were performed using the Chi-squared test. Hardy-Weinberg equilibrium was calculated using the available web-tool (Rodriguez et al., 2009).

## Results

In total, 1024 general population DNA samples and 244 depression patient DNA samples were included into the analysis. The age in the population sample was 53.8 ± 7.0 years (mean ±*SD*), the male to female ratio was 1:1. The age of depression patients was 49.0 ± 11.4 years, the male to female ratio was 1:4, with most (224) having recurrent MDD (or single episode MDD) in addition to 20 cases of dysthymia. The individuals in both study samples were of European descent.

**FIGURE 1:**
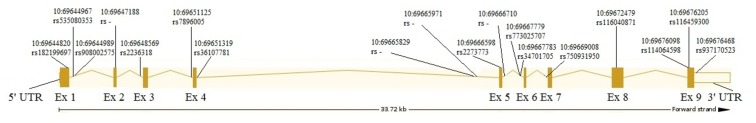
Distribution of genetic variants in the *SIRT1* gene.

The analysis revealed 20 SNPs in the *SIRT1* gene (Table 1 and Figure 1). The frequencies of the 6 most common SNPs are presented in Table 2, and those of the less frequent ( < 1%) SNPs are presented in Table 3. For the latter, only heterozygote carriers were found, which is not surprising for rare SNPs. Six rare SNPs were found only in the general population, four SNPs were found only in DD patients, and four SNPs were found in both groups.

**Table 1:** A list of the revealed SNPs in *SIRT1* gene.

Region	Position in human genome (GRSh37 assembly)	Base change	Change type	rs
Intergenic	10:69624180	G > A	Intergenic variant	rs12415800
Intergenic	10:69624232	G > A	Intergenic variant	rs7075506
Exon 1	10:69644820	C > A	Missense	rs182199697
Intron 1	10:69644967	C > G	IVS	rs535080353
Intron 1	10:69644989	G > T	IVS	rs908002575
Exon 2	10:69647188	C > A	Missense	No
Intron 2	10:69648569	T > A	IVS	rs 2236318
Intron 3	10:69651125	A > G	IVS	rs 7896005
Intron 4	10:69651319	T > C	IVS	rs36107781
Intron 4	10:69665829	A > G	IVS	No
Intron 4	10:69665971	C > T	IVS	No
Exon 5	10:69666598	T > C	Synonymous	rs 2273773
Intron 5	10:69666710	A > G	IVS	No
Intron 5	10:69667779	insATAT	IVS	rs773025707
Intron 5	10:69667783	T > C	IVS	rs34701705
Intron 6	10:69669008	T > C	IVS	rs750931950
Exon 8	10:69672479	G > A	Missense	rs116040871
Exon 9	10:69676098	C > T	Synonymous	rs114064598
Exon 9	10:69676205	A > G	Missense	rs116459300
3′ UTR	10:69676468	A > G	3′ UTR variant	rs937170523


**Table 2:** The most frequent *SIRT1* allele frequencies in Russian population (Ò = 1024) and Depressive Disorder (DD) patients (*N* = 244).

SNP genome position (rs)	10:69624232 (rs7075506)			10:69648569 (rs 2236318)			10:69651125 (rs 7896005)			10:69666598 (rs 2273773)		10:69624180 (rs12415800)		10:69651319 (rs36107781)	
						
Genotype	%GG	%GA	%AA	%TT	%TA	%AA	%AA	%AG	%GG	%TT	%TC	%GG	%GA	%TT	%TC
General population	9.1	44.4	46.5	**19.8**	**54.4**	25.8	**10.4**	**46.3**	43.3	85.1	14.9	94.1	5.9	**98.2**	**1.8**
DD patients	9.4	44.3	46.3	**37.3**	**33.6**	29.1	**16.0**	**37.3**	46.7	85.7	14.3	93.9	6.1	**96.3**	**3.7**

*Statistically significant differences (P < 0.05) are in bold.*

**Table 3:** The less frequent *SIRT1* allele frequencies in Russian population (*N* = 1024) and Depressive Disorder (DD) patients (*N* = 244).

SNP genome position (rs)	10:69644820 (rs182199697)		10:69644967 (rs535080353)		10:69644989 (rs908002575)		10:69647188 (rs-)		10:69665829 (rs-)		10:69665971 (rs-)		10:69666710 (rs-)	
							
Genotype	%CC	%CA	%CC	%CG	%GG	%GT	%CC	%CA	%AA	%AG	%CC	%CT	%AA	%AG
General population	99.8	0.2	99.7	0.3	99.9	0.1	99.9	0.1	100.0	0.0	100.0	0.00	99.9	0.1
DD patients	100.0	0.0	99.6	0.4	100.0	0.0	100.0	0.0	99.6	0.4	99.6	0.4	100.0	0.0

**SNP genome position (rs)**	**10:69667779 (rs773025707) insATAT**		**10:69667783 (rs34701705)**		**10:69669008 (rs750931950)**		**10:69672479 (rs116040871)**		**10:69676098 (rs114064598)**		**10:69676205 (rs116459300)**		**10:69676468 (rs937170523)**	
							
**Genotype**	**%wt**	**%ins**	**%TT**	**%TC**	**%TT**	**%TC**	**%GG**	**%GA**	**%CC**	**%CT**	**%AA**	**%AG**	**%AA**	**%AG**

General population	100.0	0.0	**100.0**	**0.0**	99.9	0.1	99.9	0.1	99.9	0.1	99.9	0.1	99.9	0.1
DD patients	99.6	0.4	**99.2**	**0.8**	100.0	0.0	100.0	0.0	100.0	0.0	99.6	0.4	100.0	0.0

*Statistically significant differences (P < 0.05) are in bold.*

Of the 20 SNPs, 4 were identified for the first time; therefore, their rs-numbers are not assigned yet and are not indicated in Table 3. These SNPs were positioned at 10:69647188, 10:69666710, 10:69665829, and 10:69665971.

The occurrences of homozygote carriers for two SNPs – T/T for rs2236318 and A/A for rs7896005 – were significantly higher in DD than in general population (*P* < 0.001 and *P* < 0.05, respectively). The occurrences of heterozygote carriers for these two SNPs were significantly lower (*P* < 0.0001 and *P* < 0.05, respectively). The occurrences of heterozygote carriers of two additional, less frequent SNPs – rs36107781 and rs34791705 – were significantly higher in DD than in general population (*P* < 0.05). However, analysis of Hardy-Weinberg equilibrium for these SNPs in the sample of general population revealed equilibrium only for rs2236318 (χ^2^ = 8.62, *P* < 0.005).

The allelic frequencies of the SNPs presented in Table 2 are similar to those indicated in the “1000 Genomes” program for European population (data taken from^2^; Table 4).

**Table 4:** Allele frequencies of the most frequent *SIRT1* SNPs in general Russian population (*N* = 1024) and in Depressive Disorder (DD) patients (*N* = 244) in comparison with the “1000 genomes” study (data for European population).

Position in Human genome	rs	Group	Minor allele frequency (in the current study), %	Minor allele frequency in “1000 genomes,” %
10:69624180	rs12415800	Population	2.97	2.3
		DD patients	3.07	
10:69624232	rs7075506	Population	68.66	66.9
		DD patients	68.44	
10:69648569	rs2236318	Population	53.03	52.8
		DD patients	45.90	
10:69651125	rs7896005	Population	66.44	65.0
		DD patients	65.37	
10:69651319	rs36107781	Population	0.91	1.5
		DD patients	1.84	
10:69666598	rs2273773	Population	7.48	7.6
		DD patients	7.17	

## Discussion

Comprehensive NGS analysis of the *SIRT1* structure in the general population DNA samples (*N* = 1024) and in DD patient DNA samples (*N* = 244) revealed 20 SNPs (Table 1). Among those SNPs were 4 new genetic variants not described earlier (10:69647188, 10:69665829, 10:69665971, and 10:69666710). Significant differences between these two groups were found in the frequency of heterozygous carriers for rs2236318, rs7896005, rs36107781 and rs34791705 (Tables 2, 3), supported by the Hardy-Weinberg equilibrium in the sample of general population for the first SNP (rs2236318). However, no difference was observed in the allele frequencies (Table 4).

Our results indicate that three frequent *SIRT1* genotypes (rs2236318 genotype T/T, rs7896005 genotype A/A and rs36107781 genotype T/C) may be associated with DD, with the rs2236318 genotype T/T identified as having a *P*-value < 1 × 10^-4^. The absence of Hardy-Weinberg equilibrium for rs7896005 and rs36107781 in the sample of general population does not allow ascertaining those SNPs that are associated with DD. However, almost perfect Hardy-Weinberg equilibrium for rs2236318 (*p* < 0.005) and a high prevalence of genotype T/T among DD patients (*p* < 0.001) demonstrate a strong association of this SNP with DD.

To our knowledge, these associations have been identified for the first time. Two previous studies demonstrated a relationship between DD and *SIRT1* rs12415800 allele frequencies in Han Chinese melancholic DD women (CONVERGE Consortium, 2015) and – to a much smaller degree – in an European self-reporting depression sample (Hyde et al., 2016) coded as rs187810158 in that study. In contrast to those studies, we did not find such a difference in rs12415800 (GA heterozygotes 6.1% vs. 5.9%; Table 2). Most likely, it is due to some differences among the samples (ethnicity, gender, difference in diagnostic procedures). Our patients represented both sexes with clinically verified DSM-IV-based diagnoses of depression, mainly ‘classical’ depression, with melancholic features (78%). Moreover, both those studies (CONVERGE Consortium, 2015; Hyde et al., 2016) were focused mainly on the allele frequencies and not on the occurrence of homozygous/heterozygous allele carriers.

Several rare SNPs (2 cases of rs34701705 and 1 case each of rs773025707, 10:69665829, and 10:69665971) were found exclusively in DD patients, which may indicate a possible correlation of these variants with this disorder. Larger cohort studies are needed for verifying these associations.

Most SNPs found in this study are mapped to the introns, although 4 SNPs result in missense mutations: rs182199697 (C > A) results in Pro114Gln, 10:69647188 (C > A) results in Phe148Leu, rs116040871 (G > A) results in Glu536Lys, and rs116459300 (A > G) results in Asn700Ser. Two other SNPs caused synonymous changes (rs114064598 and rs2273773) and one SNP (rs937170523) was mapped to the 3′-UTR (Table 1 and Figure 1).

There are two limitations for this study: (1) while the study suggests an important role for *SIRT1* in DD, it should be noted that this disorder represents a polygenic phenotype and that many other loci remain to be discovered beyond *SIRT1*; (2) the quality of experienced early environment (e.g., parental care, exposure to childhood abuse) was not evaluated in the DD group. Thus, one should keep in mind that the behavioral phenotype of DD featuring worse depressive symptomatology, may putatively be associated with *SIRT1* allelic variants. At least, the recent data on a reduction in the levels of the deacetylase *SIRT1* in peripheral blood mononuclear cells in this type of DD patients, significantly predicting the extent of behavioral despair, is giving credibility to this suggestion (Lo Iacono et al., 2015).

As the Novosibirsk population is similar to that of Russia, the *SIRT1* SNPs frequencies obtained in this study may be generalized to the entire population of European descent in Russia.

Overall, our preliminary findings suggest that the revealed DD-linked *SIRT1* SNPs might confer susceptibility to this disorder in Russian population of European descent, thereby extending growing body of data on *SIRT1* as a novel DD risk gene.

## Ethics Statement

All studies were approved by local Ethics Committees, and the signed informed consent included the permission from the patients to perform a genetic analysis of their DNA samples. The local Ethics Committees are: (1) Committee for Biomedical Ethics of the Institute of Physiology and Basic Medicine, (2) Ethics Committee of the Institute of Preventive and Internal Medicine (now incorporated into the Institute of Cytology and Genetics, Novosibirsk), and (3) Ethics Committee of the Mental Health Research Institute (now incorporated into the Tomsk National Research Medical Center of the Russian Academy of Sciences).

## Author Contributions

The study was conceived and supervised by LA, SK, and KD. DNA samples of DD patients were provided by LA, KD, NV, NB, and SI. Population DNA samples were provided by VM and MV. The genetic analysis was done by MA, DB, AG, and SK. The sequence data analysis and statistics were made by MA. The results were discussed and manuscript was written by LA, SK, and KD. All authors approved the final version of the manuscript.

## Conflict of Interest Statement

The authors declare that the research was conducted in the absence of any commercial or financial relationships that could be construed as a potential conflict of interest.

## Footnotes

1 http://www.ucl.ac.uk/easteurope/hapiee.html

2 https://www.ncbi.nlm.nih.gov/variation/tools/1000genomes
